# Effects of Methylsulfonylmethane (MSM) on exercise-induced oxidative stress, muscle damage, and pain following a half-marathon: a double-blind, randomized, placebo-controlled trial

**DOI:** 10.1186/s12970-017-0181-z

**Published:** 2017-07-21

**Authors:** Eric D. Withee, Kimberly M. Tippens, Regina Dehen, Deanne Tibbitts, Douglas Hanes, Heather Zwickey

**Affiliations:** Helfgott Research Institute, National University of Natural Medicine, 2220 SW 1st Ave, Portland, OR 97201 USA

**Keywords:** Methylsulfonylmethane, Exercise, Oxidative stress, 8-hydroxy-2′-deoxyguanosine, Musculoskeletal Pain, Malondialdehyde, Creatine Kinase, Lactate Dehydrogenase

## Abstract

**Background:**

Oxidative stress and muscle damage occur during exhaustive bouts of exercise, and many runners report pain and soreness as major influences on changes or breaks in training regimens, creating a barrier to training persistence. Methylsulfonylmethane (MSM) is a sulfur-based nutritional supplement that is purported to have pain and inflammation-reducing effects. To investigate the effects of MSM in attenuating damage associated with physical exertion, this randomized, double-blind, placebo-controlled study evaluated the effects of MSM supplementation on exercise-induced pain, oxidative stress and muscle damage.

**Methods:**

Twenty-two healthy females (*n* = 17) and males (*n* = 5) (age 33.7 ± 6.9 yrs.) were recruited from the 2014 Portland Half-Marathon registrant pool. Participants were randomized to take either MSM (OptiMSM®) (*n* = 11), or a placebo (*n* = 11) at 3 g/day for 21 days prior to the race and for two days after (23 total). Participants provided blood samples for measurement of markers of oxidative stress, and completed VAS surveys for pain approximately one month prior to the race (T_0_), and at 15 min (T_1_), 90 min (T_2_), 1 Day (T_3_), and 2 days (T_4_) after race finish. The primary outcome measure 8-hydroxy-2-deoxyguanine (8-OHdG) measured oxidative stress. Secondary outcomes included malondialdehyde (MDA) for oxidative stress, creatine kinase (CK) and lactate dehydrogenase (LDH) as measures of muscle damage, and muscle (MP) and joint pain (JP) recorded using a 100 mm Visual Analogue Scale (VAS). Data were analyzed using repeated and multivariate ANOVAs, and simple contrasts compared post-race time points to baseline, presented as mean (SD) or mean change (95% CI) where appropriate.

**Results:**

Running a half-marathon induced significant increases in all outcome measures (*p* < 0.001). From baseline, 8-OHdG increased significantly at T_1_ by 1.53 ng/mL (0.86–2.20 ng/mL CI, *p* < 0.001) and T_2_ by 1.19 ng/mL (0.37–2.01 ng/mL CI, *p* < 0.01), and fell below baseline levels at T_3_ by −0.46 ng/mL (−1.18–0.26 CI, *p* > 0.05) and T_4_ by −0.57 ng/mL (−1.27–0.13 CI, *p* > 0.05). MDA increased significantly at T_1_ by 7.3 μM (3.9–10.7 CI, *p* < 0.001). Muscle damage markers CK and LDH saw significant increases from baseline at all time-points (*p* < 0.01). Muscle and joint pain increased significantly from baseline at T_1_, T_2_, and T_3_ (*p* < 0.01) and returned to baseline levels at T_4_. Time-by-treatment results did not reach statistical significance for any outcome measure, however, the MSM group saw clinically significant (Δ > 10 mm) reductions in both muscle and joint pain.

**Conclusion:**

Participation in a half-marathon was associated with increased markers of oxidative stress, muscle damage, and pain. MSM supplementation was not associated with a decrease from pre-training levels of oxidative stress or muscle damage associated with an acute bout of exercise. MSM supplementation attenuated post-exercise muscle and joint pain at clinically, but not statistically significant levels.

## Background

Participation in group exercise events has risen dramatically over the past twenty years. The number of Americans finishing organized running races has risen from approximately 4.2 million in 1990 to over 15.5 million in 2012 [1]. The total active membership of USA Triathlon has grown from just under 65,000 in 1994 to over half a million in 2012 [2]. This trend is indicative of an increase in exhaustive training programs. Intermediate runners training for a half-marathon run an average of 25–30 miles per week, and can often exceed 35 miles per week in the three months before the race [3].

Exhaustive exercise increases oxygen consumption and stimulates mitochondria and lymphocytes, resulting in the production of reactive oxygen and nitrogen species (RONS) [4, 5]. At low to moderate levels, RONS are critical for cellular regulation of gene expression and cell signaling [6]. In muscle cells, they act as signals for cell adaptation and are thus necessary for muscle growth [7]. At high levels they become destructive to tissues, damaging cells and inhibiting muscle growth [8]. Oxidative stress or damage occurs when RONS react with cellular constituents and deform the constituent to an unusable form [9].

Two primary groups of biological constituents damaged by oxidative stress are DNA bases and fatty acids [9]. High levels of RONS become genotoxic when in contact with DNA. Guanine has the lowest oxidation potential of the four bases and thus is most commonly oxidized, forming 8-hydroxy-2′- deoxyguanosine (8-OHdG). When the body repairs the damage 8-OHdG is removed and excreted, becoming a marker for the oxidative stress. This damage, although possible in most tissues, occurs primarily in muscle tissue and circulating lymphocytes. DNA damage occurring as a result of oxidative stress may play an important role in the etiology of cancer, diabetes, and arteriosclerosis [10]. Lipids within the cell membrane are also damaged when high levels of RONS are present in the body. Oxidation of fatty acids in the membrane result in lipids degrading into malondialdehyde (MDA) [11]. MDA is a mutagenic byproduct and reacts with deoxyadenosine and deoxyguanosine in DNA, forming DNA adducts, and is itself damaging to the body, sometimes causing cell lysis and cell death [12]. Breaking of muscle cells releases creatine kinase (CK) and lactate dehydrogenase (LDH) into the blood stream – two enzymes found at high levels in muscle cells [8, 13]. In this study, 8-OHdG, a measure of oxidative stress, was chosen as the primary outcome measure. The secondary measure of MDA also measured oxidative stress. The secondary outcomes of muscle damage were measured through cellular enzymes (CK and LDH), and muscle pain and joint pain were measured using a visual analogue scale (VAS).

Physical training adapts the body to higher levels of RONS and increases its ability to manage the excess oxidative potential. However, long bouts of exercise still produce an unmanageable amount of oxidative stress. It has been suggested that the relationship between health and exercise is U-shaped, with negative health outcomes present both at low and very high levels of exercise [14]. Many negative health outcomes associated with very high levels of exercise are caused by excessive oxidative stress on the tissues. These outcomes include increased muscle fatigue, lower force production, and delayed onset muscle soreness (DOMS) [6, 13].

Methylsulfonylmethane (MSM) is a small sulfur-based molecule comprised of a sulfur atom with two double-bonded oxygen atoms and two methyl groups. It is a naturally occurring molecule found in a variety of foods including fruits, vegetables, grains and milk. Although it is most commonly supplemented to improve joint health, recent studies indicate that MSM may also be effective in seasonal allergic rhinitis, interstitial cystitis, autoimmune disease and cancer chemoprevention [15]. Studies have demonstrated that it has both anti-inflammatory and antioxidative properties [16, 17]. Although MSM, when ingested, has an antioxidative effect in vivo, the molecule itself does not directly quench oxidant activity. Instead, MSM reduces the body’s production of RONS [18]. It takes repeated doses over at least one week for MSM to build to an efficacious level within the body. This characteristic comes from its quick absorption rate and slower excretion rate [19].

In animal models, MSM has been shown to bolster the antioxidative capacity of cells compared to placebo controls, suggesting the presence of protective physiological pathways. In one study, MSM significantly decreased malondialdehyde (MDA), myeloperoxidase (MPO), and IL-1β, while increasing glutathione (GSH) and catalase (CAT) production [20]. Another study showed that MSM pre-treatment significantly attenuated damaging effects of acetaminophen including decreased GSH and superoxide dismutase (SOD), and increased MDA and MPO [21]. Using monocrotaline-induced pulmonary hypertensive rats, one study found significantly lower levels of CAT, SOD, and GSH in the hypertensive rats, and these levels recovered to control levels in MSM-treated rats [16].

Initial human exercise studies have suggested that MSM supplementation may reduce exercise-induced oxidative stress and muscle damage. In untrained males, supplementation of 50 mg/kg/day reduced levels of MDA, PC, and oxidized glutathione after an acute bout of exercise, when compared to placebo [22], and 50 mg/kg/day MSM supplementation increased the total antioxidant capacity (TAC) and reduced CK and bilirubin levels after exhaustive exercise compared to placebo [23]. Another study evaluating moderately-trained men noted reduction in muscle soreness and fatigue with increased MSM dosage, while trolox equivalent antioxidant capacity (TEAC) increased and homocysteine decreased at 3.0 g/day [24]. These studies suggest that MSM may bolster pathways that protect against excessive oxidative stress from exercise.

These studies evaluated untrained and moderately-trained men. The effects of MSM on trained individuals and women is still unknown. Determining MSM’s effectiveness on trained individuals is important because it is the population to most likely benefit from reduced oxidative and muscle damage. In addition, the current study was designed to observe the effects of MSM in a real-world application. We hypothesized that supplementing MSM at 3 g/day for three weeks reduces the oxidative damage induced by a bout of exhaustive exercise, and that the reduction of oxidative stress results in reduced muscle damage and subsequently reduces muscle soreness and joint pain.

## Methods

### Study participants

Included participants were in good health and between the ages of 21 and 45; all participants were recruited from the registrant pool of the Portland Half Marathon. Participants were excluded if they had taken MSM in the past month, warfarin, corticosteroids or statins in the past three months, used tobacco in the past six months, were pregnant or nursing, or if they were peri- or postmenopausal. In order to reduce variation in training regimens, competition in a race of greater than a 5 K within the month prior to the race was considered a basis for exclusion. Potential participants were excluded if they had ever participated in a full marathon, participated in a half-marathon during previous six months, or participated in more than three half-marathons during previous three years. Previous race history was used to exclude highly trained individuals and provide a study population of participants who could be considered “weekend warriors.”

Potential participants were screened for initial eligibility via a telephone call and then scheduled for a baseline visit. At the baseline visits, participants gave informed consent and then completed an eligibility and health questionnaire, a training log capturing training information for three prior days, and listed medication and supplement use. This study was approved by the National University of Natural Medicine Institutional Review Board.

### Experimental design

This study was a randomized, double-blind, placebo-controlled trial evaluating the effects of MSM on oxidative stress, cell damage and pain caused by exhaustive exercise. Twenty-four participants were randomized to receive either MSM or placebo at a dose of 3 g/day for three weeks preceding the Portland Half-Marathon. Blood serum and pain-scale questionnaires were collected approximately one month prior to the race day (baseline), and again 15 min, 90 min, 1 day and 2 days post-race. Serum samples were analyzed for the primary outcome measure 8-hydroxy-2′-deoxyguanosine (8-OHdG), and malondialdehyde (MDA); both are markers of oxidative stress. Serum levels of creatine kinase (CK) and lactate dehydrogenase (LDH) were measured as markers of muscle damage. A 100 mm Visual Analog Scale (VAS) was used to measure muscle pain and joint pain at each time point.

### Interventions

Participants ingested either 3 g/day of MSM or 3 g/day of rice flour placebo. This study used OptiMSM® provided by Bergstrom Nutrition (Vancouver, WA) as the active supplement. Placebo capsules contained rice flour, a harmless and inert substance used in food products, and not known to affect RONS. Placebo capsules, also provided by Bergstrom Nutrition, were matched for size and color to MSM capsules.

All study participants were registered for, and completed the Portland Half-Marathon (13.1 miles) held on October 5th, 2014. The start and finish lines for the race were both in downtown Portland. Decisions on training regimens, diet, and competitive goals were left to individual participants. The study did not collect pre-race training or dietary information. Participants completed a 24 h recall form for alcohol consumption at each time point, but were only asked to abstain from alcohol between the 15 min and 90 min post-race visits.

Participants took MSM or placebo starting three weeks prior to the race and continued until two days afterward. Study participants were provided with printed supplementation instructions and a capsule intake log to help track and promote supplement adherence. Participants received a reminder phone call the day before beginning supplementation and mid-study phone calls to check for adherence and adverse reactions. All participants were asked to return the remaining capsules and intake log at the final visit, as well as to complete an exit questionnaire to track any changes in participant weight or medication and supplement use.

### Outcome measures

Participants provided blood samples and completed a pain questionnaire at each study visit. Baseline visits (T_0_) occurred approximately one month prior to race day, and were conducted at Helfgott Research Institute (HRI) at NUNM. The 15 min (T_1_) and 90 min (T_2_) post-race visits occurred near the finish line of the Portland Half-Marathon in fully equipped study tents. The 1 day (T_3_) and 2 day (T_4_) post-race visits occurred at HRI at various times between 8:00 am and 7:00 pm. All blood draws were performed by a licensed phlebotomist, physician, or registered nurse. Blood collection at each visit consisted of a venous blood draw of approximately 8 to 11 mL into two separate serum separator tubes (SST): a 3.5 mL yellow top BD Vacutainer®, and an 8.5 mL “tiger top” BD Vacutainer®. Tubes were labeled with stickers containing the participant ID number and the visit number.

Blood samples were allowed to clot for 30 min at room temperature and then centrifuged at 2500 rpm for 30 min. Serum from the 8.5 mL tubes was transferred to 1.5 mL microcentrifuge tubes in 500 μL aliquots and stored at −80 °C in the HRI Laboratory for analysis of 8OHdG and MDA. The 3.5 mL tubes were stored at 4 °C overnight and transported the following day to the NUNM Clinic Laboratory where they were submitted to MESA Labs (Portland, OR) for analysis of Creatine Kinase and Lactate Dehydrogenase.

Serum 8-OHdG levels, the primary outcome measure, were measured through competitive ELISA kits purchased through Cayman Chemical (DNA/RNA Oxidative Damage EIA Kit: Item Number 589320). Assays were performed in the Helfgott Research Center Laboratory using serum aliquots from 8.5 mL SST stored at −80 °C. ELISAs were performed according to manufacturer’s instructions and absorbance was measured at 450 nm. Serum MDA levels were measured through a colorimetric assay purchased through Cayman Chemical (TBARS Assay Kit: Item Number 10009055). Assays were performed in the Helfgott Research Center Laboratory using serum aliquots from the 8.5 mL SST stored at −80 °C. Assays were conducted according to manufacturer’s instructions and absorbance was measured at 530 nm. Serum CK and LDH levels were measured by spectrophotometry at MESA Labs using serum from the 3.5 mL SST.

A 100 mm Visual Analog Scale was used to quantify perceived joint pain and perceived muscle pain by measuring, in millimeters, the distance between the left end of the scale (no pain) to the participant’s mark. Lowest possible score was 0, the highest was 100. For measurements of pain it is important to determine a clinically significant difference, which is the difference or change in scores for which a patient or participant is able to distinguish between pain levels. Clinical significance for the 100 mm VAS pain scale has been determined to be a difference of 10 mm or more (>Δ10mm) [25, 26].

### Statistical analysis

SPSS statistical software was used for all statistical analyses [27]. Initial analysis included independent t-tests for between group differences in BMI, age, gender, and race time, to check for possible differences between groups. Repeated ANOVAs were used to assess time and time*group effect. Data was assessed for normal distribution and corrections were made when appropriate. Correlations were analyzed for “Hours from Race Finish” and 1-Day and 2-Day outcomes. Multivariate ANOVAs were used to assess differences between groups at each time point. We tested unadjusted differences in means, group effects adjusted for baseline levels, and effects adjusted for baseline, BMI, Age, and Race Time, in a three-tiered analysis. Summary estimates are reported as mean ± standard deviation (SD) for timepoint analysis, and for between-group analysis, means are reported with 95% confidence intervals (CI). Comparison data for both timepoint and group analysis are reported with 95% confidence intervals.

Power calculations were completed for 8-OHdG as the primary outcome measure. Although little prior research had been conducted on serum 8-OHdG measured through ELISA, the number of necessary participants for each group was calculated at 16 (32 total) for the study to be powered at 0.80 with α = 0.05 [28, 29]. To account for a 25% dropout rate, the study set a recruitment goal of 44 participants. Data are presented as mean (SD) or mean change (95% CI) when appropriate.

## Results

**Fig. 1:**
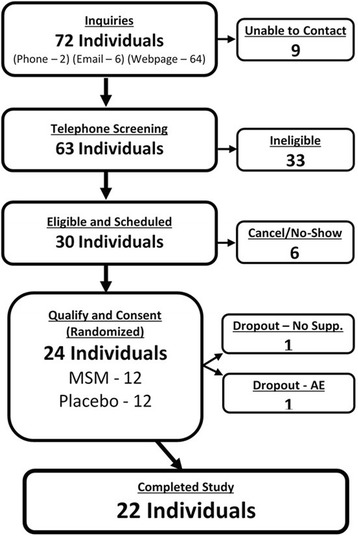
Recruitment and Enrollment. Flow chart for participant recruitment and enrollment

Recruitment did not reach the threshold required by a priori power calculations. Twenty-four healthy females and males were enrolled in the study and 22 completed all study activities (Table 1). Two participants dropped out prior to study completion: one participant dropped out due to an adverse event and one due to time constraints (Fig. 1). One participant (F, MSM Group) did not complete pain questionnaires for T_1_ and T_2_. These missing data points were left blank and no values were imputed. We were unable to obtain a blood sample from one participant (F, Placebo Group) at T_1_. Data values for blood serum measures at this one data point represent the mean of 5 imputations using a linear regression imputation model. Analyses of CK results were adjusted with a logarithmic transformation to correct for high levels of skewness. Analysis of pre-baseline training logs did not show any significant associations with baseline outcome measures.

**Table 1:** Participant Characteristics

Variable		Group (n)		
		MSM(11)	Placebo(11)	Total(22)
Gender	F	9	8	17
	M	2	3	5
		Mean ± SD	Mean ± SD	Mean ± SD
Age (years)		35.6 ± 6.5	31.8 ± 7.1	33.7 ± 6.9
BMI		25.6 ± 6.7	27.6 ± 7.6	26.6 ± 7.1
Race Time (min)		145 ± 25	162 ± 55	153 ± 43

### Pain outcomes

Running a half-marathon was associated with significant increases in both muscle pain (*p* < 0.001) and joint pain (*p* < 0.001), after controlling for baseline pain and group allocation. Mean levels of both muscle and joint pain significantly increased from baseline at T_1_, T_2_, and T_3_, but returned to non-significant levels at T_4_ (Table 2).

**Table 2:** Post-exercise outcomes compared to baseline

Outcome	N		T_0_ (Base)	T_1_ (15 Min.)	T_2_ (90 Min.)	T_3_ (1 Day)	T_4_ (2 Day)
8OHdG (ng/mL)	22	Mean ± SD	9.06 ± 3.28	10.59 ± 4.00	10.25 ± 3.57	8.60 ± 2.98	8.50 ± 2.50
		Δ_Baseline_		1.53** (0.86–2.20)	1.19* (0.37–2.01)	−0.46 (−1.18–0.26)	−0.57 (−1.27–0.13)
MDA (μM)	22	Mean ± SD	17.4 ± 4.7	24.7 ± 7.7	18.3 ± 6.1	16.2 ± 4.3	17.3 ± 4.7
		Δ_Baseline_		7.3** (3.9–10.7)	0.9 (−1.8–3.6)	−1.2 (−2.9–0.5)	−0.1 (−2.2–1.9)
logCK (U/L)	22	Mean ± SD	1.99 ± 0.20	2.25 ± 0.26	2.32 ± 0.28	2.58 ± 0.46	2.35 ± 0.42
		Δ_Baseline_		0.26** (0.13–0.38)	0.33** (0.20–0.46)	0.59** (0.39–0.79)	0.36** (0.17–0.54)
LDH (U/L)	22	Mean ± SD	158.2 ± 18.9	230.4 ± 41.3	232.4 ± 48.0	188.4 ± 31.9	180.1 ± 35.1
		Δ_Baseline_		72.2** (54.6–89.8)	74.2** (53.9–94.6)	30.2** (17.0–43.4)	21.9* (8.1–35.7)
Muscle Pain (mm)	21	Mean ± SD	15.3 ± 20.6	38.6 ± 25.6	33.6 ± 27.6	36.2 ± 25.3	21.4 ± 21.1
		Δ_Baseline_		23.3** (11.6–35.1)	18.3* (5.7–31.0)	20.9** (9.3–32.5)	6.2 (−3.5–15.9)
Joint Pain (mm)	21	Mean ± SD	8.7 ± 19.3	33.5 ± 21.3	31.2 ± 25.2	23.9 ± 23.2	16.4 ± 26.2
		Δ_Baseline_		24.8** (15.0–34.6)	22.5** (10.9–34.0)	15.2* (4.6–25.9)	7.7 (−4.3–19.7)

Results adjusted for mean-centered baseline. Means reported with standard deviations. Δ_Baseline_ indicates the change from baseline (T_0_) – reported with 95% confidence interval. *P < 0.01 vs baseline. **P < 0.001 vs baseline

**Fig. 2:**
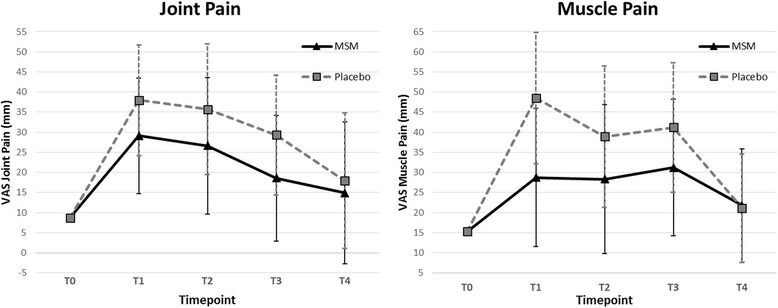
Muscle and Joint Pain. Reported pain levels (in millimeters of VAS) at each time point for Muscle pain and Joint pain. Each graph shows mean outcomes for placebo and MSM groups at each timepoint. Error bars indicate 95% confidence intervals. Results were controlled for baseline

Baseline pain levels were non-significantly lower in the MSM group for both muscle and joint pain. The results showed a pattern of lower pain levels in the MSM group (Fig. 2). However, time-by-treatment outcomes did not reach significance in either muscle or joint pain. Compared to placebo, MSM supplementation resulted in non-significantly lower muscle pain at T_1_ (*p* = 0.063), and lower muscle pain at T_2_ and T_3_. The levels of reduction in the MSM group at T_1_, T_2_, and T_3_ were at levels considered clinically significant (clinical significance defined as Δ > 10 mm) [25, 26], but none reached statistical significance. Similar results were seen for joint pain at T_3_ (Table 3).

**Table 3:** Between-group comparisons

Outcome	Group	N	T_1_ (15 Min.)	T_2_ (90 Min.)	T_3_ (1 Day)	T_4_ (2 Day)
8OHdG (ng/mL)	MSM	11	10.87 (9.90–11.83)	10.72 (9.55–11.89)	9.12 (8.09–10.15)	9.13 (8.13–10.13)
	Placebo	11	10.32 (9.36–11.29)	9.79 (8.61–10.96)	8.08 (7.05–9.11)	7.86 (6.86–8.86)
	Δ_Group_		0.54 (−0.84–1.92)	0.94 (−0.74–2.61)	1.04 (−0.43–2.51)	1.27 (−0.17–2.71)
MDA (μM)	MSM	11	26.6 (21.7–31.4)	18.8 (14.9–22.6)	15.7 (13.3–18.1)	15.9 (13.0–18.8)
	Placebo	11	22.9 (18.1–27.7)	17.9 (14.0–21.7)	16.7 (14.3–19.2)	18.7 (15.8–21.6)
	Δ_Group_		3.7 (−3.2–10.5)	0.9 (−4.6–6.4)	−1.1 (−4.5–2.4)	−2.8 (−6.9–1.4)
logCK (U/L)	MSM	11	2.26 (2.08–2.43)	2.33 (2.15–2.52)	2.67 (2.38–2.95)	2.40 (2.14–2.66)
	Placebo	11	2.24 (2.07–2.41)	2.30 (2.12–2.48)	2.49 (2.21–2.78)	2.29 (2.03–2.55)
	Δ_Group_		0.02 (−0.23–0.26)	0.03 (−0.23–0.29)	0.18 (−0.24–0.58)	0.11 (−0.26–0.48)
LDH (U/L)	MSM	11	224.3 (199.2–249.3)	228.3 (199.3–257.2)	191.0 (172.2–209.7)	183.1 (163.4–202.7)
	Placebo	11	236.5 (211.5–261.5)	236.6 (207.6–265.5)	185.8 (167.0–204.5)	177.1 (157.5–196.7)
	Δ_Group_		−12.2 (−47.8–23.4)	−8.3 (−49.5–32.9)	5.2 (−21.5–31.9)	6.0 (−22.0–33.9)
Muscle Pain (mm)	MSM	11	28.7 (11.6–45.9)	28.3 (9.9–46.8)	31.2 (14.3–48.2)	21.8 (7.6–35.9)
	Placebo	10	48.5 (32.2–64.9)	38.9 (21.2–56.5)	41.2 (25.0–57.3)	21.1 (7.6–34.6)
	Δ_Group_		−19.8 ^a^ (−43.7–4.1)	−10.6 ^a^ (−36.3–15.2)	−10.0 ^a^ (−33.5–13.7)	−0.7 (−20.4–19.1)
Joint Pain (mm)	MSM	11	29.1 (14.7–43.5)	26.6 (9.6–43.6)	18.6 (2.9–34.2)	14.9 (−2.8–32.5)
	Placebo	10	38.0 (24.2–51.7)	35.7 (19.5–51.9)	29.3 (14.4–44.2)	17.9 (1.1–34.7)
	Δ_Group_		−8.9 (−29.1–11.3)	−9.1 (−33.0–14.7)	−10.7 ^a^ (−32.7–11.2)	−3.0 (−27.8–21.7)

Results adjusted for mean-centered baseline. Group results reported as Mean (95% confidence interval). Δ_Group_ indicates between group differences at each timepoint (MSM – Placebo), results reported with 95% confidence interval

^a^Clinically significant (Δ_Group_ ≥ 10) betweeen-group pain outcomes

### Serum outcomes

The MSM group had lower baseline levels of 8-OHdG and MDA and higher levels of CK and LDH, although none of these differences were statistically significant. All serum marker calculations were adjusted using the mean-centered baseline level of the outcome measure as a covariate during analysis.

**Fig. 3:**
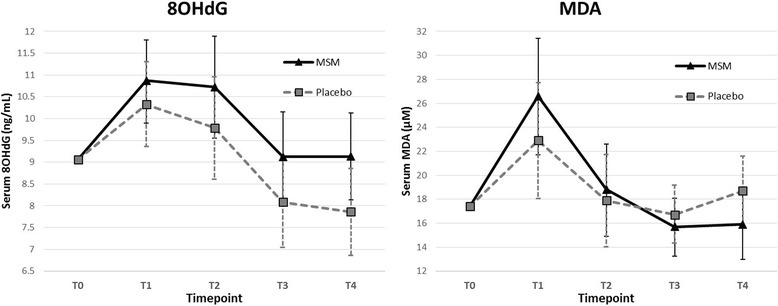
Oxidative Stress. Serum oxidative stress levels, measured in levels of 8-OHdG and MDA. Each graph shows mean outcomes for placebo and MSM groups at each timepoint. Error bars indicate 95% confidence intervals. Results were controlled for baseline

Running a half-marathon induced significant increases in oxidative stress in both 8-OHdG (*p* < 0.001) and MDA (*p* < 0.001) immediately after the race (Times T_1_ and T_2_), when controlling for baseline and group (Fig. 3). Mean 8-OHdG serum levels fell to levels below baseline measurements at T_3_ and T_4_. Mean MDA serum levels across treatment groups increased significantly from baseline levels directly after the race at T_1_, but recovered to levels similar to baseline by T_2_ (Table 2). When adjusted for baseline, time-by-treatment outcomes for both 8-OHdG and MDA were insignificant. A consistently higher level of 8-OHdG in the MSM group was observed, and the mean difference in 8-OHdG levels between MSM and Placebo increased over time (Fig. 3). MDA levels in the MSM group were higher at T_1_ and T_2_, but fell below Placebo group levels at T_3_ and T_4_. Between-group differences in MDA were non-significant at each time point (Table 3).

**Fig. 4:**
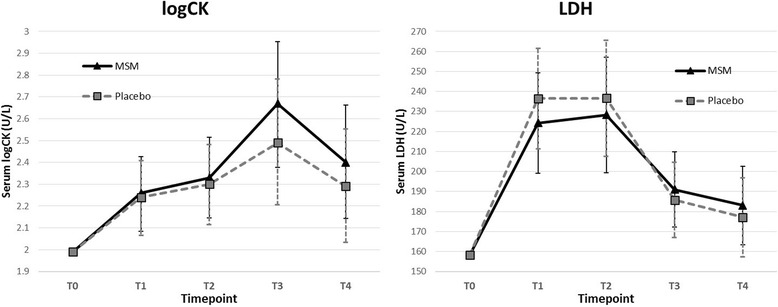
Muscle Damage. Serum levels of LDH and logarithmically adjusted CK – indicators of muscle damage. Each graph shows mean outcomes for placebo and MSM groups at each timepoint. Error bars indicate 95% confidence intervals. Results were controlled for baseline

Running a half-marathon significantly increased levels of both log-transformed CK (logCK) (*p* < 0.001) and LDH (*p* < 0.001) when controlling for baseline and group (Fig. 4). Mean serum levels of logCK significantly increased (*p* < 0.001) from baseline at every post-race time point, with T_4_ levels being highest. Mean LDH serum levels also increased significantly from baseline levels at all post-race time points, with the similarly high levels measured at T_1_ and T_2_. LDH levels at T_3_ and T_4_ fell to levels significantly (*p* < 0.001) lower than at T_1_ and T_2_ (Table 2). When adjusted for baseline, time-by-treatment outcomes for both logCK and LDH were insignificant. Log adjusted CK levels were nearly identical between groups at T_1_ and T_2_. At T_3_ and T_4_ a measurable, yet non-significantly higher level of logCK was observed in the MSM group compared to placebo (Fig. 4). The MSM group had lower levels of LDH at T_1_ and T_2_, but higher levels than Placebo at T_3_ and T_4_. Group differences were non-significant at each time point (Table 3).

### Adverse events and side effects

Two participants dropped out of the study. One participant in the MSM group dropped out due to a change in employment that restricted availability prior to beginning supplementation. One participant in the Placebo group dropped out due to self-reported adverse reactions after one week of supplementation that included low-energy and difficulties concentrating.

No other adverse events were reported during the study. Upon exit, two participants in the MSM group reported experiencing mild gastrointestinal effects and one reported experiencing insomnia. No side effects were reported in Placebo group.

## Discussion

Muscle and joint pain levels were lower in the MSM group at all post-race time points for both muscles and joints, although overall group comparisons were non-significant. Between group time comparison for muscle pain at 15 min did result in a statistical trend (*p* = 0.063) for lower pain in the MSM group. It is important to note that although treatment did not result in statistical significance, the size of the differences between the MSM and Placebo groups at 15 min, 90 min and one day were clinically significant (Δ > 10 mm). A reduction of 10 mm or greater is generally accepted as clinically relevant [25, 26]. The reduction of 19.8 mm for muscle pain, although not statistically significant, is bolstered by its clinical relevance. Between-group outcomes for joint pain resulted in clinically significant lower pain levels of between 9 and 10 mm. Although the results did not reach statistical significance, the clinically significant effect combined with similar results across two pain measures suggests that MSM may be effective in reducing exercise-induced pain.

While no between-group comparisons reached statistical significance at any time point, there was a statistical trend for lower 8-OHdG levels in the Placebo group two days after the race. The differences in 8-OHdG between MSM and Placebo groups that occurred at one and two days after the race appear to have occurred in part due to Placebo group levels falling below baseline. Treatment appeared to have almost no effect on the muscle damage markers CK and LDH. Logarithmically adjusted CK levels were slightly higher in the MSM group at each time point, but differences were minimal and statistically equivalent. Group comparisons for LDH showed slightly lower levels in the MSM group for race day time points, and slightly higher levels in the one and two day time points.

This study aimed at recruiting a group of participants who are not year-round competitive runners, but rather people who have decided to enter a race as a means of increasing their exercise for personal accomplishment, or for social benefits. By having a group of participants who were not overly trained, we hoped to create a group for which running a half-marathon would induce a measurable oxidative stress response. Because the body adapts to exercise, a group of highly trained individuals may not have a strong oxidative stress response to running a distance that is regularly achieved. Overall, the recruitment criteria were effective in creating a study group reflective of this larger population. A key element in this success was recruiting from a pool of people who already opted-in to running the race. The study group was slightly overweight, had an average age of 35 years, and completed the race in just over two and a half hours, on average. Body mass index was similar to U.S. population, age was similar to all half-marathon participants, and race time was slightly higher than overall U.S. average times – reflective of first or second attempts [1, 30]. A higher number of female runners is typical in distance running, but our study did not include enough men to reasonably test for potential gender differences in outcome measurements. However, because women are an underrepresented group in studies of this type, we consider the high number of female participants in our study population to be a strength of our study.

Three previous studies evaluating the effects of MSM on untrained men showed significant reductions in the oxidative stress markers MDA and protein carbonyls, a reduction in the muscle damage markers CK and bilirubin, as well as an increase in total antioxidant capacity (TAC) and glutathione [22, 23, 31]. These studies used a similar exercise intervention (14 km run) as the present study. Another clinical trial showed MSM supplementation at 3 g/day increased trolox equivalent antioxidant capacity (TEAC) in moderately trained males after completing knee extensions [24]. One possible explanation for this discrepancy is differing study populations. The study population was comprised of moderately trained participants. It is possible that the ability of MSM to attenuate exercise-induced oxidative stress and muscle damage in untrained and moderately-trained men does not work in broader populations, including women, populations with higher BMIs, and varying training levels. In addition, knee extensions have a very different effect on physiological pathways than running a half-marathon [24].

Interestingly, the results of this study suggest that MSM supplementation may simultaneously reduce muscle and joint pain, yet increase oxidative stress markers. It also suggests a correlation between higher levels of oxidative stress and muscle pain (data not shown). Although statistical significance was not reached in the group comparisons, overall patterns seem to place these findings at odds. This discrepancy may have resulted from the lack of a true baseline measurement. Baseline levels were measured approximately one month prior to the race, representing a pre-training measurement rather than a pre-race measurement. A pre-training baseline measurement is not able to account for varying training regimens, which have a considerable impact on oxidative stress responses. A measurement either the night before or immediately before the race would have provided a better representation of a pre-race baseline level. A baseline blood-draw was not taken directly before the race due to logistical constraints and the excessive burden it would have placed on the participants.

If the discrepancy did not result from a lack of true baseline, MSM must reduce pain through a separate mechanism of action. Several studies have indicated that MSM may have anti-inflammatory properties. MSM has been shown to reduce levels of NF-κβ, IL-1, IL-6, IL-8, TNF-α, and the activation of inflammasomes [17, 32, 33]. It is possible that the reduction in pain stems from a reduction in inflammation.

The body adapts to repetitive exercise, including attenuation of oxidative stress [5]. This is especially true for habitual exercise routines [34], and it is common for the intensity of training regimens to be highest the month prior to the race [3]. Differences in training, individual response to training, and level of individual athleticism in the month prior to the race can have major impacts on normal levels of oxidative stress and muscle damage [6, 35]. Oxidative stress levels in this study were lower at one and two days after the race than at baseline, indicating a biochemical adaptation occurred during the month between baseline and the race. Furthermore, previous research gives no evidence that training regimens affect normal, or baseline pain levels. Therefore the baseline pain measurements, unlike the serum measurements, were likely similar at the start of the race to those measured one month prior, and resulted in changes more reflective of previous research.

### Limitations

Although the group was largely reflective of the targeted population, males were underrepresented and recruitment did not meet goals for number of participants (Fig. 1). The low number (*n* = 5) of male participants prohibited adjusting for gender in statistical analysis, as the low representation would make results highly unreliable. The original aim was to have 32 participants finish the study as per the power calculations conducted on 8-OHdG. Recruitment was hindered by an unexpectedly high percentage of race participants residing in areas not amenable to study involvement. Fortunately, a strong study design and effective incentives reduced the drop-out rate and maximized data capture. It was assumed that 25% of recruited participants would drop from the study. The study ended with a dropout rate of 8% (2/24).

8-OHdG was chosen as the primary outcome measure because of its novelty as a serum marker of exercise-induced oxidative stress. In prior exercise studies, it was more common to use urine 8-OHdG levels, while serum markers have more commonly been used in studies analyzing food or drug impacts on oxidative stress. Although investigating unique markers can result in a deeper understanding of the mechanism of action, the lack of prior research has a limiting effect on study design and implementation. Furthermore, analysis of 8-OHdG was performed through competitive ELISA, which introduced inter-assay variability. This was addressed in part by ensuring that all time points from a single individual were assayed together, thus removing any inter-assay variability from time point changes.

## Conclusions

The study suggests that MSM supplementation may attenuate exercise-induced muscle and joint pain at clinically relevant levels, but that it does not reduce post-exercise markers of oxidative stress or muscle damage from pre-training levels. We also observed that running a half-marathon reliably induces muscle and joint pain, oxidative stress and muscle damage, over a predictable time course. It still remains uncertain whether MSM supplementation attenuates exercise-induced oxidative stress and muscle damage from pre-race levels, or whether results observed in previous research only apply to narrow populations. Further research on the effects of MSM on pre-exercise levels of oxidative stress and muscle damage, and research examining the effects of MSM supplementation on post-exercise pain among a larger sample of broadly-trained participants is warranted.

## Abbreviations

8-OHdG: 8-hydroxy-2′-deoxyguanosine
CAT: Catalase
CK: Creatine kinase
DOMS: Delayed onset muscle soreness
GSH: Glutathione
HRI: Helfgott Research Institute
LDH: Lactate dehydrogenase
MDA: Malondialdehyde
MPO: Myeloperoxidase
MSM: Methylsulfonylmethane
NUNM: National University of Natural Medicine
PC: Protein carbonyls
RONS: Reactive oxygen and nitrogen species
SOD: Superoxide dismutase
TAC: Total antioxidant capacity
TEAC: Tolox equivalent antioxidant capacity
VAS: Visual analog scale
